# Supplementary material for: Development of a fasting blood glucose-based strategy to diagnose women with gestational diabetes mellitus at increased risk of adverse outcomes in a COVID-19 environment
Source: PLoS One. 2020 Dec 3;15(12):e0243192. doi: 10.1371/journal.pone.0243192 (PMC7714128; doi:10.1371/journal.pone.0243192)
Supplement: Attachment — Submitted filename: PLOS_Rebuttal-Reviewer.pdf [file pone.0243192.s002.pdf]

Queensland Government

Royal Brisbane & Women's Hospital
Enquiries to: Dr. Michael d'Emden
Endocrinology & Diabetes
Telephone: 3646 7955
Facsimile: 3646 7089

31 July 2020

Frank T. Spradley
Academic Editor
PLOS ONE

Thank you for reviewing our paper titled :-

"Development of a fasting blood glucose-based strategy to diagnose women with gestational diabetes mellitus at increased risk of adverse outcomes in a COVID-19 environment".

PONE-D-20-26829

We provide the following responses to the points raised by the reviewer.

We have modified line 5 of the abstract so that is now reads POGTT for the diagnosis of gestational diabetes mellitus (GDM). We felt "for the diagnosis" was better wording than "in the diagnosis".

ii) Line 10. "Higher risk" has been changed to "high risk".

iii) We have removed "real life study".

iv) We have corrected references 2 and 14 and made minor corrects to several other references as well as updating some links

00201:61107

Office	Postal	Phone	Fax
Level 1, East Block<br>Brisbane Q 4029	Post Office<br>Brisbane Q 4029 Australia	07 3646 7955<br>ISD + 61 7 3646 7955	07 3646 7089

We have included the analysis of the UK Covid -19 diagnostic approach to GDM which is based, in part, on an HbA1c of 5.7% that results in 81% of cases of GDM being missed. We think that this clearly illustrates that an HbA1c is not a good screening test for GDM. It now reads:-

An HbA1c has not proved that useful for the diagnosis of GDM as opposed to overt diabetes [6, 17]. This was clearly demonstrated in the analysis of the UK recommendations for diagnosis of GDM during the COVID-19 pandemic where a diagnostic strategy based in part on an HbA1c > 5.7% (39 mmol/mol) resulted in 81% of cases fulfilling IADPSG criteria being missed [4].

We trust that these modifications and additions address the issues raised by the reviewer and we thank him/her for their time and thoughts.

Yours sincerely

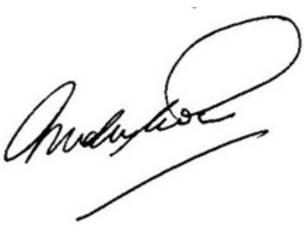

Dr Michael d'Emden

00201:61107

Royal Brisbane and Women's Hospital – we don't smoke here anymore

**Office**	**Postal**	**Phone**	**Fax**
Level 1, East Block<br>Brisbane Q 4029	Post Office<br>Brisbane Q 4029 Australia	07 3646 7955<br>ISD + 61 7 3646 7955	07 3646 7089
